# The Role of Negative Charge in the Delivery of Quantum Dots to Neurons

**DOI:** 10.1177/1759091415592389

**Published:** 2015-07-20

**Authors:** Ryan Walters, Igor L. Medintz, James B. Delehanty, Michael H. Stewart, Kimihiro Susumu, Alan L. Huston, Philip E. Dawson, Glyn Dawson

**Affiliations:** 1 Committee on Neurobiology, University of Chicago, IL, USA; 2 Center for Bio/Molecular Science and Engineering, Code 6900, U.S. Naval Research Laboratory, Washington, DC, USA; 3 Optical Sciences Division, Code 5611, U.S. Naval Research Laboratory, Washington, DC, USA; 4 Scripps Research Institute, La Jolla, CA, USA; 5 Departments of Pediatrics, Biochemistry and Molecular Biology, University of Chicago, IL, USA

**Keywords:** charge, neuron, quantum dot, endosomal uptake, oligodendrocyte, chondroitin sulfate

## Abstract

Despite our extensive knowledge of the structure of negatively charged cell surface proteoglycans and sialoglycoconjugates in the brain, we have little understanding of how their negative charge contributes to brain function. We have previously shown that intensely photoluminescent 9-nm diameter quantum dots (QDs) with a CdSe core, a ZnS shell, and a negatively charged compact molecular ligand coating (CL4) selectively target neurons rather than glia. We now provide an explanation for this selective neuronal delivery. In this study, we compared three zwitterionic QD coatings differing only in their regions of positive or negative charge, as well as a positively charged (NH_2_) polyethylene glycol (PEG) coat, for their ability to deliver the cell-membrane-penetrating chaperone lipopeptide JB577 (WG(Palmitoyl)VKIKKP_9_G_2_H_6_) to individual cells in neonatal rat hippocampal slices. We confirm both that preferential uptake in neurons, and the lack of uptake in glia, is strongly associated with having a region of greater negative charge on the QD coating. In addition, the role of negatively charged chondroitin sulfate of the extracellular matrix (ECM) in restricting uptake was further suggested by digesting neonatal rat hippocampal slices with chondroitinase ABC and showing increased uptake of QDs by oligodendrocytes. Treatment still did not affect uptake in astrocytes or microglia. Finally, the future potential of using QDs as vehicles for trafficking proteins into cells continues to show promise, as we show that by administering a histidine-tagged green fluorescent protein (eGFP-His_6_) to hippocampal slices, we can observe neuronal uptake of GFP.

## Introduction

**Figure 1.**
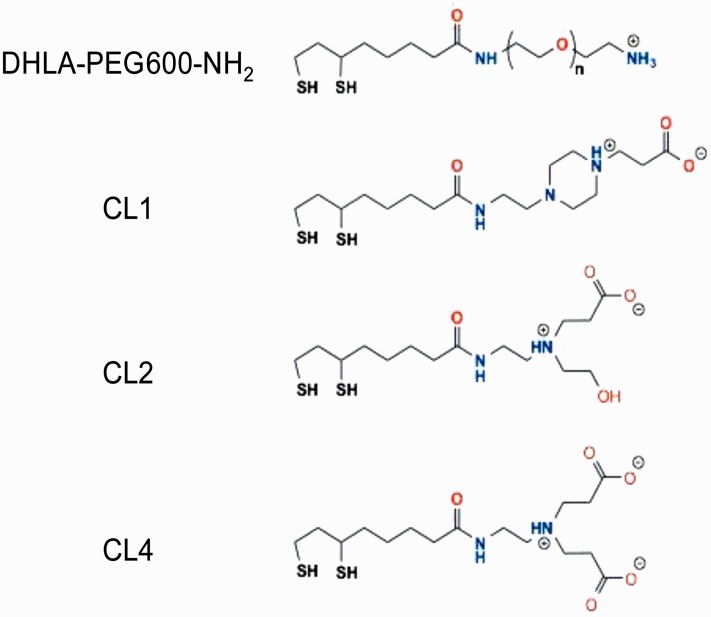
QD surface functionalization ligands or coatings. Structure of compact ligand (CL) coatings CL1, CL2, and CL4 as well as the PEG (DHLA-PEG600-NH_2_, *n* = 12) used in the studies. DHLA-PEG600-NH_2_ has an overall positive charge from the amine. CL1 has potential positive charges from the piperazine in addition to negative charge from the carboxyl group. CL2 has one positively charged nitrogen and one negatively charged carboxyl and is probably zwitterionic under physiological conditions. CL4 has one positively charged nitrogen and two negatively charged carboxyls at the terminal end. QD = quantum dot; PEG = polyethylene glycol; DHLA = dihydrolipoic acid.

Storage disorders can be treated by enzyme replacement therapy unless they involve the central nervous system (CNS; Hobert and Dawson, 2007; Jalanko and Braulke, 2009). For example, we are unable to treat late infantile neuronal ceroid lipofuscinosis (LINCL), a lysosomal storage disorder in which a mutation in *CLN2* (11p15) causes neurons to fail to produce enzymatically viable tripeptidylpeptidase 1 (TPP1) and store the subunit c of mitochondrial ATP synthase. This is due in part to the difficulty in trafficking proteins across the blood–brain barrier as well as failure to target specific cell types, such as neurons. Delivering bioactive peptides to subcellular compartments and cytosol of specific cell types of the CNS and to visually track that delivery and subsequent fate of the cargo is our goal, and we previously reported the use of quantum dots (QDs) to deliver peptides to neurons in rat hippocampal slices (Walters et al., 2012). The commonly used approach of fluorophore attachment to a peptide of interest must preserve reliable fluorescence through tissue application, fixation, and viewing, which can lead to photobleaching and subsequent loss of signal (Algar et al., 2011); use of QDs can overcome many of these problems (Delehanty et al., 2010; Boeneman et al., 2013). Peptides alone, with or without targeting signals conjugated to them, will rarely pass the blood–brain barrier, and not in any concentration that would be efficacious (Carman et al., 2011). The use of semiconductor QDs provides a potential vehicle for delivery, bioactivity testing, and visualization of attached cargos due to their robust fluorescence, resistance to photobleaching, and ease of peptide attachment via utilizing a simple polyhistidine-Zn interaction (Medintz et al., 2005). Native QDs such as those synthesized with a CdSe/ZnS core/shell structure need to be surface functionalized to make them colloidally stable and reduce toxicity (Susumu et al., 2007, 2011); this also allows researchers to append peptides to the QD surface to assist in trafficking or peptide ligation. In this study, we have used several modified compact ligand (CL) coatings (Figure 1) that show greater cell-type specificity of uptake than those displaying neutral or unmodified polyethylene glycol (PEG). We have previously shown that zinc on the surface of the QD can bind histidine-tagged peptides and proteins such as maltose-binding protein (Boeneman et al., 2013), and that by using the QD as a delivery vehicle, we can attach signaling peptides to the surface to further specify the target location. We previously showed that a QD with a negative coat (CL4) facilitated the delivery of a cargo (Palm-1/JB577) to neurons rather than astrocytes, oligodendrocytes, or microglia (Walters et al., 2012) and now report that the surface charge is critical for this cell-specific uptake.

In the current study, we expand our QD investigation to include three different CL coats with modified structures and resultant regions of charge and found that the coats varied dramatically in their targeting behavior. In addition, we utilize a positively charged PEG coating and show uptake in oligodendrocytes. Finally, we modified the charge of the extracellular matrix (ECM) of two *in vitro* systems and found that this can also contribute to QD targeting (Soleman et al., 2013). These results confirm that both the charge of the QD, as well as the charge of the target cell, strongly influences the trafficking and fate of the QD in the CNS and by inference the intercellular trafficking of proteins.

## Materials and Methods

### Quantum Dots

CdSe/ZnS core/shell QDs with an emission maxima centered at ∼625 nm were made hydrophilic by exchanging the native hydrophobic ligands with dihydrolipoic acid (DHLA)-based CLs shown in Figure 1, see also Susumu et al. (2011). In addition, a positively charged DHLA-PEG-amine (DHLA-PEG600-NH_2_) ligand was also used. This displayed a terminal amine group instead of the neutral methoxy group used in our first study (Walters et al., 2012).

### Peptides

The palmitoylated peptide (JB577) sequence used was AcWG(Pal)DapVKIKKP_9_GGH_6_, where *Pal* corresponds to a C16:0 palmitate group that is covalently attached to a nonhydrolysable thiol-resembling diaminopropionic acid residue (Dap) functionality synthesized into the peptide backbone (Sapsford et al., 2007). All peptides were synthesized using Boc (t-butoxycarbonyl)-solid phase peptide synthesis, purified by high-performance liquid chromatography (HPLC), and purity verified by electrospray ionization-MS (ESI-MS; Dawson et al., 2010). All peptide sequences are written in the conventional N- to C-terminal orientation. The peptides were purified, desalted, and quantitated before being lyophilized and stored at −20℃ until used as described (Sapsford et al., 2009).

### Microscopy and Image Analysis

The intracellular distribution of QDs was analyzed by fluorescence microscopy using a Marianas fully automated Yokogawa-type spinning disc confocal microscope equipped with 40 × and 100 × oil-immersion lenses. Quantification of fluorescence via pixel intensity was measured through ImageJ and graphically represented through Microsoft Excel. A Student’s independent *t* test was performed on all quantification with a significance of *p* ≤ .005.

### Peptide-Mediated QD Delivery

QD–JB577 bioconjugates were formed by diluting a stock solution of JB577 with QD at room temperature (1 µM QD assembled with 1, 10, or 100 JB577 peptides per QD in 0.1 M borate buffer, pH 8.9). After 15 min of conjugation, the conjugate was administered into a complete growth medium overnight to a final QD concentration of 50 to 100 nM. These peptide:QD ratios were determined experimentally for the peptide to be the ratio that yielded the optimal degree of uptake without overexpression, and 1:25 QD:peptide is used for these experiments unless otherwise noted.

### Uptake by Rat Hippocampal Slice Cultures

Hippocampal cultures were prepared as described previously (Kunkler and Kraig, 1997; Mitchell et al., 2010; Walters et al., 2012). All procedures involving animals were approved by the Institutional Animal Care and Use Committee at the University of Chicago and conducted in accordance with the guidelines of the National Institutes of Health. Briefly, Wistar rat pups (P9–P10, where P is postnatal day) were anesthetized with a progressive exposure to 100% CO_2_ and decapitated, the brains removed and placed into Hanks balanced salt solution (HBSS; 3℃) supplemented with D-glucose (6.5 mg/ml). Hippocampi were then isolated and sectioned (350-mm thick) perpendicular to their septotemporal axis. Slices displaying an intact pyramidal neuron cell layer and dentate gyrus were transferred to uncoated 30-mm Millicell-CMTM tissue culture inserts (Millipore, EMD Millipore, Billerica, MA, USA) in six-well culture dishes. Cultures were initially maintained in medium containing 23% horse serum (no. 26050–088; Invitrogen, Div. of Thermo Fisher Scientific, Grand Island, NY, USA) for 18 days *in vitro* before being transferred to serum-free media for an additional 3 days *in vitro* before experimentation (Mitchell et al., 2010). Slice cultures were used between 21 and 35 days *in vitro*. The constituents of horse-serum-based and serum-free media are defined elsewhere (Mitchell et al., 2010). Hippocampal brain slice cultures mature *in vitro* and closely resemble the anatomical and physiological characteristics of their *in vivo* counterparts (e.g., see Kunkler and Kraig, 1997, 1998, 2004; Hulse et al., 2004; Kunkler et al., 2005; Grinberg et al., 2011, 2012; Pusic et al., 2011; Walters et al., 2012).

### Uptake by Cultured Oligodendrocytes and Astrocytes

Neonatal rat oligodendrocytes were isolated from cerebral hemispheres by the Bottenstein modification of the method of McCarthy and de Vellis (1980) (Qi and Dawson, 1993). Briefly, rat oligodendrocytes and astrocytes will be separated from the rest of the brain and grown upon a polylysine-coated plate. The astrocytes will form a bed layer upon the flask, while the oligodendrocytes and remnant microglia will attach to that. The oligodendrocytes can then be separated from the astrocytes by a series of shakes. The first shake is carried out at 7 days and subsequently at 1-week intervals with shakes 2, 3, and 4 giving the highest yield of 98% pure astrocytes and microglia-free neonatal rat oligodendrocytes. All cultures were differentiated for 6 days prior to use. Finally, astrocytes are isolated from the flask through trypsinization of the bed layer (following oligodendrocyte removal by shaking) and cultured on polylysine in Dulbecco’s modified Eagle’s medium supplemented with 10% fetal bovine serum.

### Antibodies and Staining

Hippocampal slices were fixed to gel-coated slides using Prolong Gold Antifade (Invitrogen, no. P36931) containing 6-diamidino-2-phenylindole (DAPI), a nuclear stain that binds strongly to A–T-rich regions in DNA. Neuronal staining was carried out through Nissl staining with NeuroTrace 500/525 green (Invitrogen, no. N21480), which binds to negatively charged nucleic acids in the endoplasmic reticulum (ER) of neurons (Quinn et al., 1995). For oligodendrocytes, we used a mouse monoclonal antibody to myelin basic protein (MAB1580, Millipore), which has been shown to specifically stain both early and mature oligodendrocytes and myelin (Friedman et al., 1989). Staining for astrocytes is achieved with an antibody to glial fibrillary acidic protein (GFAP; Sigma; Aldrich Co. St. Louis, MO, USA, no: G3893), which is largely expressed in astrocytes, and shown to be a robust method of detection (Courel et al., 1986). Microglia were stained using isolectin b4 conjugated to Alexa Fluor 488 (Invitrogen, no. I21411), isolated from the African legume *Griffonia simplicifolia* and shown to be a robust stain for microglia (Streit, 1990). To detect digested chondroitin sulfate (CS) stubs, we used monoclonal antibody MAB2030 from Millipore Inc.

## Results

The CLs used here are based off of the DHLA moiety necessary to attach this coating to the QDs and colloidally stabilize them (Algar et al., 2011; Susumu et al., 2011). Although designed to be similarly zwitterionic, they differ in the specific regions and combinations of charges included in each and how they are structurally organized as shown in Figure 1. All three were designed to be a more compact version of DHLA-PEG ligands previously used for coating QDs (Susumu et al., 2007).

**Figure 2.**
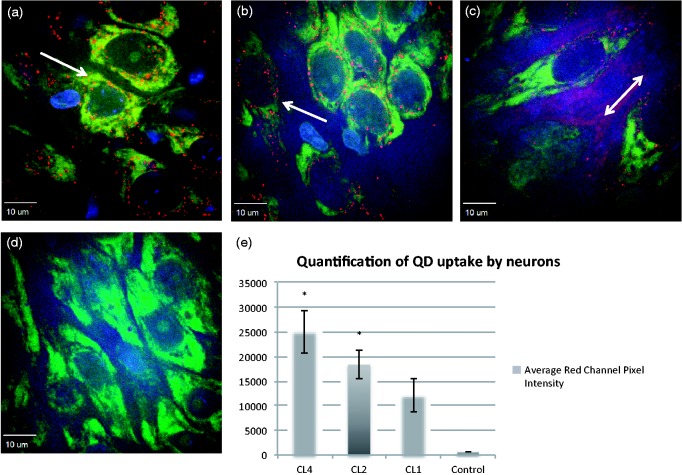
Compact ligand uptake in hippocampus. Uptake of CL4 (a), CL2 (b), and CL1 (c) by organotypic hippocampal cultures after 24-hr incubation. (a) CL4 shows robust neuronal specificity (CA3 region of the hippocampus) and no background fluorescence as we have shown previously. (b) CL2 coating shows significantly more neuronal specificity than CL1 (c) that shows less neuronal uptake as well as extensive extracellular staining, similar to our previously reported data for PEG (Walters et al., 2012). (d) Control Nissl stain without QDs. (e) Histogram showing average red channel pixel intensity for neuronal cell bodies. CL4 and CL2 are significantly more expressed in neurons than CL1 as shown by a Student’s independent *t* test (*p* ≤ .005). Red is QD, green is Nissl staining, and blue is DAPI. Arrows indicate regions of high QD expression. CL = compact ligand; QDs = quantum dots; PEG = polyethylene glycol.

**Figure 3.**
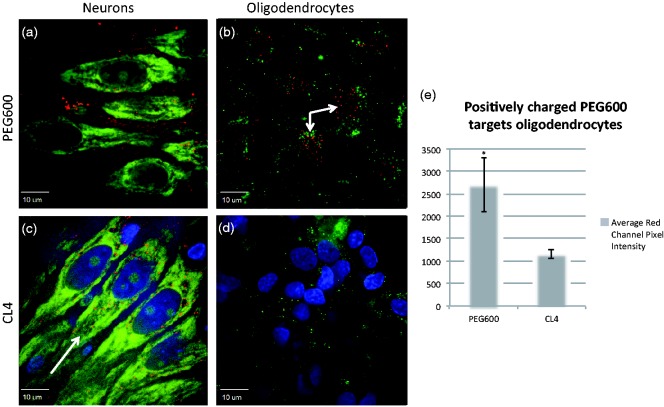
Positively charged coat traffics to oligodendrocytes in rat hippocampal slices. Positively charged PEG600-NH_2_ coat behaves similar to CL1 with regard to neurons exhibiting extracellular staining in CA3 with some neuronal trafficking. Tellingly, it appears to associate with oligodendrocytes, which is the first of the QDs tested that does so without chondroitinase treatment. (a) PEG600NH_2_ shows little specific uptake in neurons of the hippocampus with large amounts of extracellular dispersion as seen with CL1. (b) PEG600-NH_2_ traffics into oligodendrocytes with little extracellular staining indicating specificity. (c) Typical CL4 distribution within neuronal cell bodies of the hippocampus. (d) CL4 does not target oligodendrocytes, which are stained green with MAB 1580. (e) Average red channel pixel intensity of PEG600-NH_2_ and CL4 in oligodendrocytes showing over a twofold increase in trafficking with positively charged PEG600 (*p* < .005). Red represents QDs, green is cell-specific staining, and blue is DAPI. Arrows indicate regions of high QD uptake. PEG = polyethylene glycol; CL = compact ligand; QDs = quantum dots.

We have previously shown that neutral PEG-methoxy capped QDs do not give appreciable neuronal uptake, while CL4-displaying QDs show robust neuronal targeting (Walters et al., 2012). We wished to extend our previous study to include similar QDs to investigate whether charge is mediating neuronal targeting. CL1 contains a piperidine ring and only one carboxyl group, giving a region of stronger positive charge than the single nitrogen found in CL4 and CL2. CL2 could be considered neutral by virtue of the tertiary amine and the single carboxyl group, whereas CL4 has a more negatively charged region by virtue of its two carboxyl groups. We show that replacement of one carboxyl group in CL4 still allowed neuronal-specific uptake in rat hippocampal cultures (Figure 2(b) and (c)), while removing a carboxyl group and adding a piperazine ring (CL1) leads to markedly less neuronal specificity (while still maintaining some targeting), approximating what was seen with PEG (Figure 3(a)) with respect to diffuse extracellular labeling instead of strong intracellular puncta. Finally, we include a positively charged PEG QD (DHLA-PEG600-NH_2_), which exhibits similar distribution to CL1 as well as being the only QD we have tested that traffics to oligodendrocytes without treatment (Figure 3).

**Figure 4.**
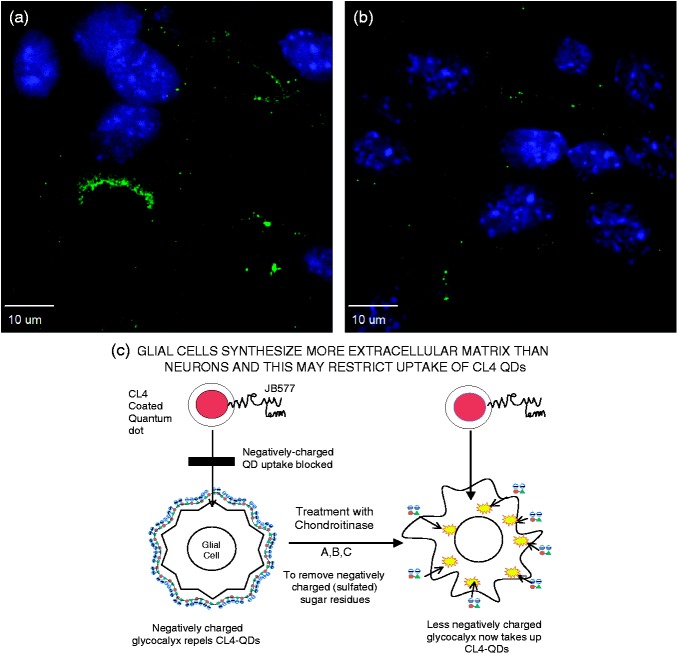
Pretreatment with chondroitinase ABC digests the extracellular matrix. Chondroitinase ABC successfully digests chondroitin sulfate chains following an overnight application in hippocampal slices. (a) Positive staining for antibody to chondroitin-4-sulfate, produced after chondroitinase digestion, shows treatment paradigm is successful. (b) Antibody staining in untreated cells (negative control). (c) Cartoon representation of the proposed action of chondroitinase ABC on the oligodendrocyte cell-membrane. CL = compact ligand; QD = quantum dot.

**Figure 5.**
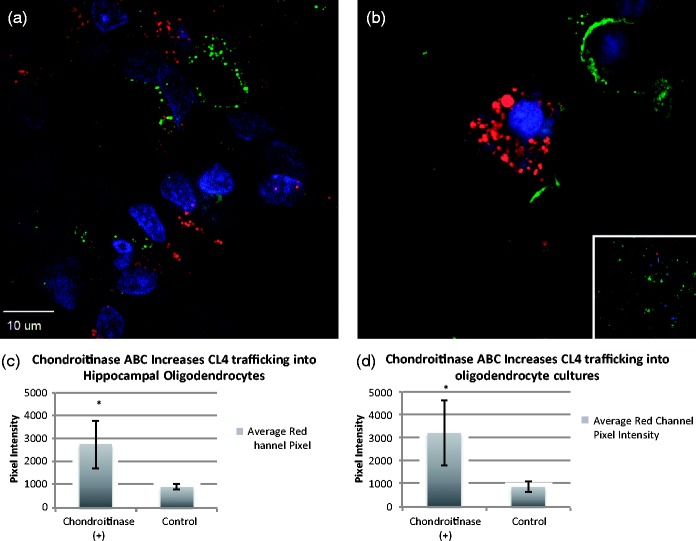
Chondroitinase ABC increases CL4 trafficking into oligodendrocytes. (a, c) 24-hr pretreatment of chondroitinase ABC (2 µM) to media of hippocampal cultures increases CL4 quantum dot uptake in oligodendrocytes. (b, d) Oligodendrocyte primary cultures after 24-hr chondroitinase ABC treatment show a similar increase in uptake (inset is an example control image of untreated culture). Red represents QDs, green is antioligodendrocyte staining (MAB 1580), and blue is DAPI. Asterisks indicate significant increase over untreated controls via Student’s independent *t* test (*p* < .005). CL = compact ligand; QDs = quantum dots; DAPI = 6-diamidino-2-phenylindole.

To determine whether the behavior of the different coats was dependent upon the milieu charge of the neuronal cultures more than the charge of the coat peptides themselves, we attempted to remove negatively charged proteoglycans (Maroto et al., 2013), expressed more heavily by glial than neuronal cells (Schwartz and Domowicz, 2014), by treating rat hippocampal slices with chondroitinase ABC. The digestion with chondroitinase ABC cleaves disaccharide units, leaving an oligosaccharide stub on the proteoglycan core protein that is recognized by a specific antibody MAB2030. This CS monoclonal antibody only reacts with digested cartilage proteoglycans, not with native proteoglycans (Takeuchi et al., 2013). Chondroitinase digestion has been shown to be somewhat efficacious in digesting glial scarring in models of spinal cord injury (Takeuchi et al., 2013). Successful digestion (Figure 4) resulted in a marked increase in oligodendrocyte uptake of CL4 QDs (Figure 5(a)) that previously had not been observed, but did not improve uptake by astrocytes or microglia.

**Figure 6.**
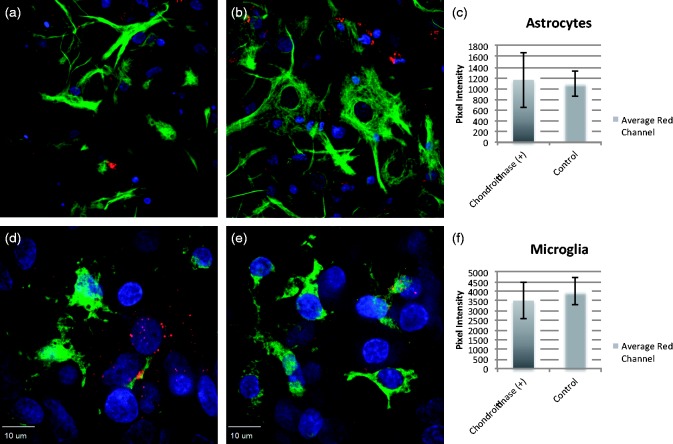
Chondroitinase ABC does not affect astrocyte or microglia trafficking. 24-hr pretreatment with chondroitinase ABC does not increase CL4 uptake in astrocyte or microglia cultures. (a) Astrocytes without chondroitinase or (b) astrocytes following chondroitinase ABC show no increase in trafficking. (c) Quantification of red channel fluorescence around positively stained cells. (d) Microglia without chondroitinase ABC or (e) microglia following chondroitinase ABC show similar results. (f) Quantification of red channel fluorescence around positively stained cells. Red represents QDs, green is cell-specific staining, and blue is DAPI. CL = compact ligand; QDs = quantum dots; DAPI = 6-diamidino-2-phenylindole.

We then used primary cultures of rat oligodendrocytes, astrocytes, and microglia to further determine the extent of chondroitinase ABC treatment on the uptake of CL4 QDs in individual cell types. Overnight treatment of oligodendrocyte cultures resulted in increased uptake of CL4 QDs (Figure 5(b)) similar to what we observed in hippocampal slices. Treatment of astrocyte or microglia cultures, however, did not lead to a significant increase in uptake (Figure 6). Therefore, there appears to be a specific interaction between the QD coat charge and the charge of the extracellular milieu of the target cell, which is enabling the neuronal specificity shown by CL4 or CL2 QDs.

**Figure 7.**
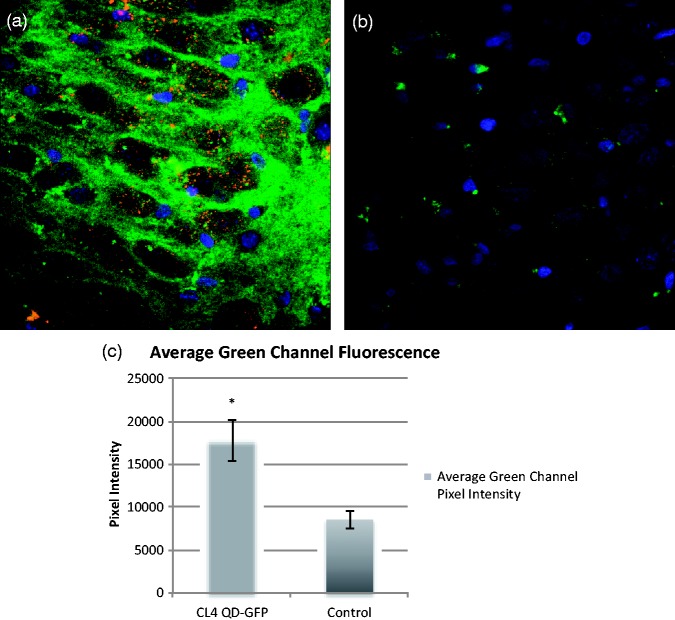
GFP can be trafficked into neurons with CL4. GFP with a histidine tag (His_6_) can be conjugated to CL4 dots and trafficked into neurons. (a) GFP-His_6_ CL4 dots were applied for 24 hr and show similar distribution in CA3 region of hippocampus as previously described for JB577-CL4 QDs (Walters et al., 2012). (b) GFP-His_6_ without CL4 shows very little uptake in the same slices when applied for the same time period. This shows that CL4 QDs can be used to traffic large proteins (32.7 kDa) such as GFP. (c) Quantification of green channel fluorescence around the CA3 region of the hippocampus following application of GFP shows that cells treated with GFP conjugated to CL4 through His_6_ show significantly increased trafficking into neurons compared with GFP alone (Student’s independent *t* test: *p* < .005). Red represents QDs, green is eGFP-His_6_, and blue is DAPI. GFP = green fluorescent protein; CL = compact ligand; QDs = quantum dots; DAPI = 6-diamidino-2-phenylindole.

Finally, to continue to the end goal of using these QDs as vehicles for protein delivery, we show that large proteins can be taken up by neurons following attachment to CL4 QDs. We conjugated a green fluorescent protein (eGFP; 32.7 kDa) with a histidine tag (His_6_) to CL4 QDs and show that this construct readily traffics into neurons (Figure 7). GFP-His_6_ CL4 QDs were applied for 24 hr and show similar distribution in the CA3 region of rat hippocampus as previously described for CL4 QDs conjugated to cell-penetrating peptide JB577 (Walters et al., 2012). In contrast, GFP-His_6_ alone showed very little uptake in the same slices when applied for the same time period showing that CL4 QDs can be used to traffic larger peptides and proteins (∼33 kDa), such as GFP, in addition to much smaller chaperone peptides such as JB577.

## Discussion

We report several findings indicating that charge plays an important role in targeting QDs to cells of the CNS. First, we show that altering the charge of the QD coating through the selective replacement of hydroxyl groups, as found on commercially available PEG QDs, with compact ligands containing negatively charged carboxyls to generate novel zwitterionic particles, significantly increases the neuronal uptake and trafficking of these dots with little to no uptake by glia. Second, to confirm the idea that charge was critical to trafficking, we show that degrading the ECM with chondroitinase ABC (an enzyme that digests negatively charged CS, a major component of the glial ECM) increased trafficking and uptake of QDs into oligodendrocytes but had no effect on astrocytes or microglia. The role of cell surface charge in cell uptake (endocytosis) has not been previously studied and represents a new concept in facilitating translational efforts in the CNS such as enzyme replacement therapy. Finally, we show that the CL4 QDs can be used to traffic proteins, in this case GFP, into the cell bodies of neurons in culture. These novel results suggest a method for directing the targeting and fate of clinically relevant cargo in the brain through the manipulation of both the vehicle’s charge as well as the charge of the target location.

Previously, we have investigated the trafficking of the novel cell-penetrating peptide WG(*Pal*)DapVKIKKP_9_G_2_H_6_ (Palm-1/JB577) conjugated to CL4 QD. QDs were chosen instead of a traditional tag due to their robust and stable fluorescence, in addition to affording stable attachment of the His6-terminated peptide to the zinc surface of the CdSe/ZnS QD. A spacer domain (Proline_9_-Glycine_2_) separates the peptide from the tag, allowing flexibility and integrity of the domains. The N-terminal tryptophan permits 280 nm absorption monitoring. The lipopeptide is a nonhydrolysable analog of the palmitoylated K-Ras4A (CVKIKK) structure in which the thiol group of cysteine was replaced by a diaminopropanol (Dap) and the remaining sequence (VKIKK) functions as a chaperone and a cell-penetrating peptide (Dawson et al., 2010; Delehanty et al., 2010).

We demonstrated that such a lipopeptide complex can be taken up into cells by endocytosis and released from the endosome over a 72-hr period (Boeneman et al., 2013). This was demonstrated in cultured cell lines by using fluorescence resonance energy transfer (FRET) analysis, which showed that the peptide initially stays associated with the QDs for at least 72 hr (Delehanty et al., 2010). Commercially available QDs are typically coated with an amphiphilic copolymer that may have additional PEG groups displayed on it to decrease toxicity as well as increase nanoparticle stability (Algar et al., 2011; Bradburne, et al., 2013). When we used our CL4 QDs instead of PEG with the JB577 peptide, we found that the QD preferentially trafficked to neurons in both rat hippocampal cultures as well as human fibroblasts (Walters et al., 2012). We observed no uptake by astrocytes and oligodendrocytes and only minimal uptake by microglia, indicating that the chemical nature of the DHLA coating may be providing neuronal specificity. In addition, we showed in hippocampal slices that there was no toxicity associated with the CdSe/ZnS QD and its cargo (negative Sytox staining and intact mitochondria; Walters et al., 2012). Santra et al. (2005) suggested that TAT-conjugated QDs, intra-arterially delivered to a rat brain labeled all brain tissue, whereas QDs without TAT did not label brain tissue, validating the idea of using a cell-penetrating peptide such as JB577 to cross the BBB. By using *in vivo* chick administration (Agarwal et al., 2015), we showed that QDs could travel from embryonic spinal cord to cell body regions of the cortex, further suggesting that JB577 may be able to facilitate transport across the blood–brain barrier.

In the current study, we continue to build upon the hypothesis that the CL4 coat is clearly mediating neuronal targeting of the CdSe/ZnS QDs. CL4 is a dipolar molecule, containing two negatively charged carboxyl groups on one end. We now report that when the QD core is coated with a DHLA-based ligand containing at least one carboxyl group as well as a tertiary amine (as in CL2), the QD can be delivered to neurons in rat hippocampal slices as efficiently as with CL4. However, a coat containing only one carboxyl group and a piperazine ring (CL1) leads to noticeably reduced neuronal specificity and a mostly extracellular distribution as we reported previously for PEG (Figure 2). Interestingly, positively charged PEG shows similar distribution as CL1 in CA3 region of the hippocampus but strikingly shows uptake in oligodendrocytes as well (Figure 3). These data indicate that charge is playing a role in the targeting of QDs into neuronal cell bodies. Such phenomena would require that charge surrounding the cells should also play a role, so we decided to investigate whether digesting negatively charged extracellular polymers such as CSs could modify the trafficking of QDs.

Extracellular factors have been shown to affect axonal regeneration, which is essential for recovery from spinal cord injury (Filous et al., 2010; Mountney et al., 2013; Takeuchi et al., 2013). CSs are produced mainly by astrocytes and oligodendrocytes in which uronic acid, plus extensive sulfation, results in a negatively charged glycocalyx (Schwartz and Domowicz, 2014). Bacterially derived enzymatic digestion of this matrix with chondroitinase ABC has been shown to increase axonal regeneration following injury and upregulation of glial-derived chondroitin sulfates proteoglycans (CSPGs) that create a barrier to axonal regrowth. A recent report (Lang et al., 2015) suggests that using a membrane-permeable peptide can block this CSPG-mediated inhibition of recovery. Similar results have been obtained by deleting N-acetylgalactosyltransferase-1 in transgenic mice (Takeuchi et al., 2013). Such a treatment or deletion does not affect heparan sulfate synthesis (in fact, it has been shown to increase it), and this proteoglycan is typically found on the surface of neurons, being important for activation of many growth factor receptors. Because endosomal uptake of CL4-QDs was observed in hippocampal neurons but not in astrocytes, oligodendrocytes, and microglia present in the same hippocampal slice, we tested the hypothesis that glial-derived, negatively charged ECM molecules such as CSs could be preventing endocytosis of QDs into nonneuronal cell types.

Chondroitinase ABC cleaves a particularly broad range of galactosaminoglycan (GalAG) substrates, including CS, dermatin sulfate (DS) and, to a much lesser extent, hyaluronic acid. Neuronal galactosaminoglycans are composed of disaccharide repeat units of uronic acid [IduA (α-L-iduronic acid) or GlcA (β-D-glucuronic acid)] (1**→**3) linked to GalNAc (N-acetyl-D-galactosamine). These basic disaccharide units are linearly associated via β-(1**→**4) linkages to form mostly polymers of CS attached at multiple points along a core protein. Biosynthesis of CS involves sulfation of the sugar backbone at various positions, which generates diversity in their oligosaccharide sequences. CS is commonly O-sulfated at the C-4 of the N-acetylgalactosamine [C4S (chondroitin-4-sulfate) or CSA], the C-6 of the N-acetylgalactosamine [C6S (chondroitin-6-sulfate) or CSC], or iduronic acid and C4 of GalNAc (CSB; Schwartz and Domowicz, 2014). Other rare modifications in CS, such as 2-O-or 3-O-sulfation of the GlcA moieties, have also been reported (Prabhakar et al., 2005). Treatment with chondroitinase ABC has been shown to cause changes in the ECM through digestion of CS and, to a much lesser extent, hyaluronan (Galtrey et al., 2007). Chondroitinase ABC digests CS-GAG chains into soluble sulfated disaccharides by beta-elimination, leaving the core protein intact (detectable by antibodies such as BE-123 to the chondroitin-4-sulfate stub). This prevents the CSPG-matrix glycoprotein interactions, which inhibit axonal regeneration (Hill et al., 2012; Lang et al., 2015). Our approach is further supported by the observation that scar formation following injury to the CNS is strongly associated with negatively charged CSs of the ECM, and treatment with chondroitinase ABC has been shown to reduce the thickness of the glial zone surrounding a cerebral artery (Hill et al., 2012). Therefore, we attempted to reduce the amount of negative charge in the ECM surrounding other cell types present in hippocampal slices through digestion of CS by chondroitinase ABC in an effort to increase uptake of CL4 QDs in these cell types.

Treatment with chondroitinase ABC for 6 hr and longer showed increased CL4 QD uptake in oligodendrocytes, both in hippocampal slice cultures and oligodendrocyte monocultures. This suggests that negative charge is a contributing factor to neuronal targeting of CL4 QDs. This argument is in agreement with our CL2 data showing similar neuronal specificity with slightly reduced uptake, and with our CL1 coat data, which shows a decrease in neuronal specificity with the removal of one of the carboxyl groups and the addition of a piperazine with two positive charges. This indicates that the areas of negative charge on the CL4 or CL2 coat convey neuronal specificity in a way that is not possible with a more positively charged coat like CL1. Together, these data show that at least one negatively charged carboxyl on the QD coat (CL2/4) is sufficient to target the QD to the neuronal cell body with little to no uptake in other cell types. By changing the charge of the coat protein from negative to positive (dipolar), however, the QD exhibits a distribution similar to what we observed using PEG QDs. Furthermore, by removal of negatively charged proteoglycans in cell culture, it is possible to increase CL4 trafficking to oligodendrocytes in both hippocampal and monoculture systems, suggesting that neuronal targeting is significantly influenced by charge.

We have previously only tested neutrally charged PEG but not positively charged nanoparticles (Walters et al., 2012). In this study, we include a positively charged variant (DHLA-PEG600-NH_2_) and show that it behaves as one would predict from our previous data with the addition that it appears to traffic nicely into oligodendrocytes in culture, something we have yet to see with the other QDs without chondroitinase treatment. Other investigators have used positively charged nanoparticles such as dimercaptosuccinate-coated iron oxide nanoparticles (D-IONPs) containing the fluorescent dye BODIPY (BP) in their coat (BP-D-IONPs). These have an overall positive charge and were shown to be preferentially taken up by oligodendroglial OLN-93 cells and not by neurons (Adams et al., 2013). Fluorescent BP-D-IONPs and nonfluorescent D-IONPs had similar hydrodynamic diameters and showed identical colloidal stability in physiological media with increasing particle size and positivation of the ζ-potential in presence of serum. Exposure of oligodendroglial OLN-93 cells to BP-D-IONPs or D-IONPs in the absence of serum increased the cellular iron content to around 1800 nmol/mg. This was reduced to around 50% at 4℃ and by around 90% by incubation in presence of 10% serum. The accumulation of both D-IONPs and BP-D-IONPs in the absence of serum was not affected by an endocytosis inhibitor. However, in the presence of serum, inhibitors of clathrin-dependent endocytosis lowered the particle accumulation by around 50%. Colocalization studies with lysotracker revealed that BP-D-IONP-containing vesicles were directed in the cells to the lysosomal pathway (Luther et al., 2013). The neurons, astrocytes, and oligodendrocytes in the slices used in our study were cultured in a synthetic serum-free medium, and cell cultures of differentiating oligodendrocytes were maintained in serum-free Bottensteins medium so the role of serum was not an issue.

Adams et al. (2013) have also shown that positively charged BP-D-IONP nanoparticles are taken up preferentially by glia. They used mouse subventricular zone-derived neural stem cells (maintained as neurospheres) for magnetofection transfection of proteins at 24 hr after plating. Transfection with Pmax-Green Fluorescent Protein:magnetic nanoparticle complexes (or pmaxGFP only for controls) was performed under four magnetic field conditions (no field, static field and oscillating fields (1 and 4 Hz)). Transfection efficiency was enhanced over twofold by 4 Hz oscillating magnetic fields and had no effect on cell viability, cell number, stem cell marker expression, or the differentiation profiles of these *magnetofected* cultures. However, only transfection of these positively charged particles into glial cells was observed, never neurons.

We therefore conclude that the surface charge on cells is another critical determinant as to whether endocytosis of QDs can take place, in addition to the charge of the QD coat. Negatively dipolar CL4 or zwitterionic CL2 will preferentially target neurons in culture, whereas neutral PEG or positively dipolar CL1 shows a much more disperse distribution. A positive QD has the inverse affect and traffics to oligodendrocytes while showing similar difficulty in associating with neurons.

A major problem in the treatment of neurodegenerative diseases is the lack of noninvasive methods of trafficking drugs to the brain. Nanoparticulate drug delivery offers the possibility of overcoming this problem through combination with a cell-penetrating peptide, better drug targeting, reduced toxicity, and improved therapeutic efficacy. The tight junctions of the blood–brain barrier cause a much higher transendothelial electrical resistance than those of other tissues, which makes intercellular space tighter and less permeable for aqueous-based paracellular transport. However, the barrier also contains specific enzyme systems, transporters, and protein receptors, which may turn out to be the key to bypassing it.
